# Epidemic Prevention During Work Resumption: A Case Study of One Chinese Company's Experience

**DOI:** 10.3389/fpubh.2020.596332

**Published:** 2021-01-21

**Authors:** Quan Zhang, Yijin Wu, Meiyu Li, Linzi Li

**Affiliations:** ^1^School of International Business and Public Management, Ocean University of China, Qingdao, China; ^2^Center for Medical Humanities in the Developing World, School of Translation Studies, Qufu Normal University, Rizhao, China; ^3^School of Economics and Management, China University of Petroleum (East China), Qingdao, China; ^4^Rizhao Maternal and Child Health Hospital, Rizhao, China

**Keywords:** COVID-19, work resumption, China, company, epidemic prevention

## Abstract

**Background:** The COVID-19 epidemic not only brings challenges to the health of people all over the world, but also impacts the global economy, and employment. Therefore, promoting industry and business to resume work safely has become an important step to be taken by all countries in overcoming the economic recession and restarting growth.

**Objective:** This study aims to elaborate on epidemic prevention measures a Chinese company (Company C) took during work resumption.

**Methods:** In this study, we used a case study design, with field research method applied to data collection and analysis.

**Results:** It has been identified that Company C took a range of measures to prevent the outbreak of COVID-19 inside the company, which involve work resumption preparation (information survey, health training, work resumption plan, epidemic prevention plan), facilities management, materials management, employee activity management, and so on.

**Conclusion:** When the COVID-19 epidemic was initially controlled in February, the Chinese government allowed enterprises to resume work voluntarily, which did not bring about a rebound in the epidemic. One important reason is that Chinese enterprises have taken multiple measures to prevent the spread of the COVID-19 virus. Company C's practices could shed some light on how companies in Western countries resume their work during the COVID-19 pandemic.

## Introduction

The COVID-19 epidemic which began in December 2019 not only poses challenges to the health of people all over the world, but has also had a negative impact on the global economy (1–4). As a developing country, the coronavirus epidemic also dealt a greater blow to China's economy. According to the national bureau of statistics, China's GDP fell 9.8% in the first quarter from a year earlier after factories, shops and travel were closed to contain the infection. This is the first time since 1979 that China has witnessed such a sudden and massive contraction in its economy (5). More than 460,000 Chinese firms closed permanently in the first quarter and more than half of them have operated for under 3 years (6).

Clearly, it is an important task for all countries to restart economic activities and promote enterprises to resume production and work. Many countries and regions began to restart economic activities and promote enterprises to resume work when the virus spread was shown to be vigorously controlled and prevented. However, the global epidemic is still severe, and enterprises still face many difficulties, the largest of which is to avoid the rebound of the epidemic caused by cluster infections occurring in the process of work resumption. In March, a significant COVID-19 cluster occurred in Smithfield Plant in South Dakota, USA, where 644 employees were infected with the COVID-19 (7). This is not an isolated case. Covid-19 clusters have also appeared in meatpacking plants around other countries including Canada, Spain, Ireland, Brazil, and Australia (8). Meatpacking plants are not the only businesses where cluster infections occurred. Also, cluster infections were reported in a parcel delivery company in Germany (9), a Brazilian petroleum company (10), and abattoirs in France (11). This conforms to the warning of Dr. Anthony Fauci, the top infectious disease expert in the United States. He claimed that pushing too quickly to reopen businesses during the COVID-19 pandemic could trigger an outbreak that you may not be able to control (12). Therefore, how to implement countermeasures to prevent cluster infections in the process of work resumption is of great significance.

When the epidemic was initially brought under control on February 10, 2020, the Chinese government began to restart the economy and allowed enterprises to resume work and production voluntarily. The resumption of production and work for Chinese enterprises did not bring about a rebound in the epidemic. Data shows that from February 10 to June 2, the number of confirmed cases and deaths caused by COVID-19 in China continued the downward trend (13). This is not only due to the prevention efforts the government at the level of both cities and villages have took, but also due to the efforts conducted by Chinese companies in the process of work resumption. The practices in the past 4 months shows that the Chinese companies' measures on preventing the epidemic during work resumption have been proven successful. These successful practices of epidemic prevention Chinese companies took could shed some light on how companies in other countries to resume their work and production.

It is an impossible task to sum up the experiences of epidemic prevention of all companies in China. Therefore, we use a case study as the main method, and obtain the research data from a typical company by field research and semi-structured interviews, which could deeply and systematically reproduce the practices of the company's measures on epidemic prevention. These measures will be approached from multiple aspects such as work resumption preparation, facilities management, materials management, and employee activity management, which could comprehensively reproduce the company's experiences concerning epidemic prevention during work resumption.

## Methods

Case research aims to understand the complexity of a demarcated entity by performing an in-depth and intensive analysis of the selected case (14, 15). Given its potential for understanding complex processes as they occur in their natural setting, case study is increasingly used in a wide range of health-related disciplines and fields, including medicine, nursing and health management (16).

### Research Question

As argued by Yin, case studies are particularly suitable for answering “how” research questions (17), our research question is in exactly how a specific company (Company C) during the COVID-19 epidemic successfully practice epidemic prevention while resuming its work and production.

### Case Selection

In this study, Company C is selected as our research case. The primary reason we choose Company C as research case is that it made great achievements on epidemic prevention during work resumption. Company C is a Sino-US joint venture and a listed company in China, located in Qingdao, China. Company C has 500 employees, and its business is spread all over the country including Hubei, Hunan, Henan, Zhejiang, and other provinces where the coronavirus epidemic is serious, this results in big challenges for epidemic prevention during the process of work resumption. The company is located in Qingdao, which is a famous tourism city where the employees travel a lot. This also brings pressure to the company's epidemic prevention. Through reasonable planning and practice, Company C has generally prevented the coronavirus epidemic in the process of work resumption, and no confirmed or suspected case occurred among the company's employees or managers. The second reason we choose company C is that it is a “typical” case. Company C not only possesses the job characteristics (task interdependence, Job autonomy, Feedback, etc.) of modern workplace (18), but also has the physical environment characteristics (factory, office building, laboratories, etc.) of manufacturing companies (19). Thus, it can be seen a typical case of modern manufacturing company. The third reason we choose company C is that one of the authors of this study is an employee of Company C, which provides convenience for us to enter the company and carry out field work and interviews during the epidemic.

### Data Collection

In case studies, researchers are encouraged to use a variety of methods to collect data in order to describe or explain a single case comprehensively and deeply (20). In this study, we use the methods of semi-structured interviews method and field research to collect data. In semi-structured interviews, semi-structured interview guidelines were used to collect qualitative data focused on the research problem (21, 22). Field research was originally an anthropological research method, and then it was expanded to other disciplines such as sociology, political science and management (23). Now, it has become a common method for collecting data during case studies (24). This method requires researchers to go into the scene and use techniques such as observation, interruption analysis and verbalizations to collect data. In observation, behavior is observed and recorded on document sheets (25). In interruption analysis, the person interrupted by the observer who ask questions about what has been previously observed. In verbalizations, participants involved in the study are asked to comment on their activities in or after the activity. Accordingly, observation, interruption analysis, and verbalizations were used to examine how the managers and employees have done to prevent the outbreak of COVID-19 inside the company.

Twelve participants are involved in the semi-structured interviews and field research and informed consent was obtained from all participants. Participants consist of 6 managers (administration department, human resources department, production department, procurement department, training and conference department, canteen department) and 6 employees (security department, cleaning department, marketing department, finance department). In observation, the participant's behavior concerning pandemic prevention was recorded on document sheets. In interruption analysis, the observer interrupted the participants to ask questions about what has been previously observed. In verbalizations, participants are asked to comment on their activities regarding epidemic prevention in or after their activities.

### Data Analysis

Data were organized and analyzed using the method of inductive content analysis. We follow scientific analysis process for the content analysis of the data based on the methodological approach by Elo and Kyngäs (26). In the preparation phase, the unit of analysis was selected. In the organizing phase, sub-categories about company epidemic prevention were identified as much as possible (27–29). Then, similar sub-categories were grouped into main categories (30). In the reporting phase, our analyzing process were reported.

To increase the trustworthiness of this study, two researchers independently coded all raw data, and coding disagreements were discussed until consensus was reached (31). In addition, during the coding process, codes were expanded and changed to ensure codes were extremely exhaustive (32). Furthermore, when the researchers reviewed the data, feedback loops were frequently used to ensure that the emerging codes, sub-themes, or themes were amended if necessary (33).

## Results

Based on our field research, it has been found that Company C's epidemic prevention measures are composed of the following themes. What need to be explained is that, there are a variety of departments in Company C, and some measures were taken at the company level, including employee information registration, health education, entrance management, public place management, dining activity management, etc., while some measures were taken at the department level. For example, productive activity management was at the workplace/factory level, cooking activity management was at the canteen level and so on.

### In Preparation for the Resumption of Work and Production

Under the pressure of economy and unemployment, the Chinese government allowed enterprises to resume work and production voluntarily on February 10, 2020. After comprehensive consideration, Company C decided to resume production and work on February 24, 2020 and made the following preparations for reopening its work and production.

#### Employee Information Report

Company C has branches all over China, including the most affected provinces such as Hubei, Hunan, Henan, Zhejiang, and so on. The sale employees of Company C made business trips to Wuhan, Huangang, Xiaogan and other epidemic-stricken cities in Hubei province during the outbreak of the epidemic, and all returned to Qingdao before the Spring Festival^1^. In addition, many employees' hometowns are outside Qingdao city or even Shandong Province, most of whom went back to their hometowns during the Spring Festival. This would increase the likelihood of their exposure to the Virus. The situations above increased the uncertainty of Company C's epidemic prevention. All employees in Company were asked to report their personal information on Tencent or WeChat platform before work resumption. All employees need to promise that all the information reported is true. Company C also keeps the information on file for future communication with the city's health administration department and emergency management department. The registered information is shown below:

1. *Personal health status: body temperature, fever, head pain, fatigue, cough, chest tightness*.
2. *Business travel information before the Spring Festival: travel time, travel route (flight/train shift), important locations arrived related to the epidemic, close contacts related to the epidemic*.
3. *Travel information during the Spring Festival: travel time, travel route, important locations arrived related to the epidemic, close contacts related to the epidemic*.
4. *Family members' travel information during the Spring Festival: travel time, travel route, important locations arrived related to the epidemic, close contacts related to the epidemic*.
5. *Personal situation when staying in Qingdao: important locations arrived related to the epidemic, close contacts related to the epidemic*.
6. *Other information related to the Covid-19 pandemic*.

Source: Company C's internal website (authorized by Company C's administrative department)

#### Flexible Work Resumption

If all employees return to work, it is difficult to maintain a safe distance between each other. Thus, Company C formulated a plan for work resumption which they called gradual and flexible work resumption. As for gradual resumption plan, core employees in all departments are asked to resume work in the company. In contrast, other employees are required to work at home. They would be allowed to return to the workplace when the epidemic is relieved. In terms of the flexible work resumption plan, the core employees asked to resume work don't have to work in the company every day. They need to work in the company only when there is work that has to be done in the company. This was a countermeasure to further reduce the staff density in the company. For employees who work at home, the company formulated an epidemic prevention plan to trace their health conditions every day, which could ensure their health and safety. An interview with the human resources manager of Company C is as follows:

*Our company's gradual and flexible resumption plan not only maintained normal operation of our company, but also ensured the health of the company's employees during the epidemic. For example, in the financial department, we arranged the accounting supervisor as the first batch of personnel to resume work in order to restart the department's business quickly and formulate the work resumption plan in financial department as soon as possible. Other employees in the financial department work at home. This plan works well. I think it is good for the company and its employees*.

*Source: Ms. Qiao, Manager of human resources department in Company C*

#### Company C's Action Plan on the Epidemic Prevention

**Figure 1:**
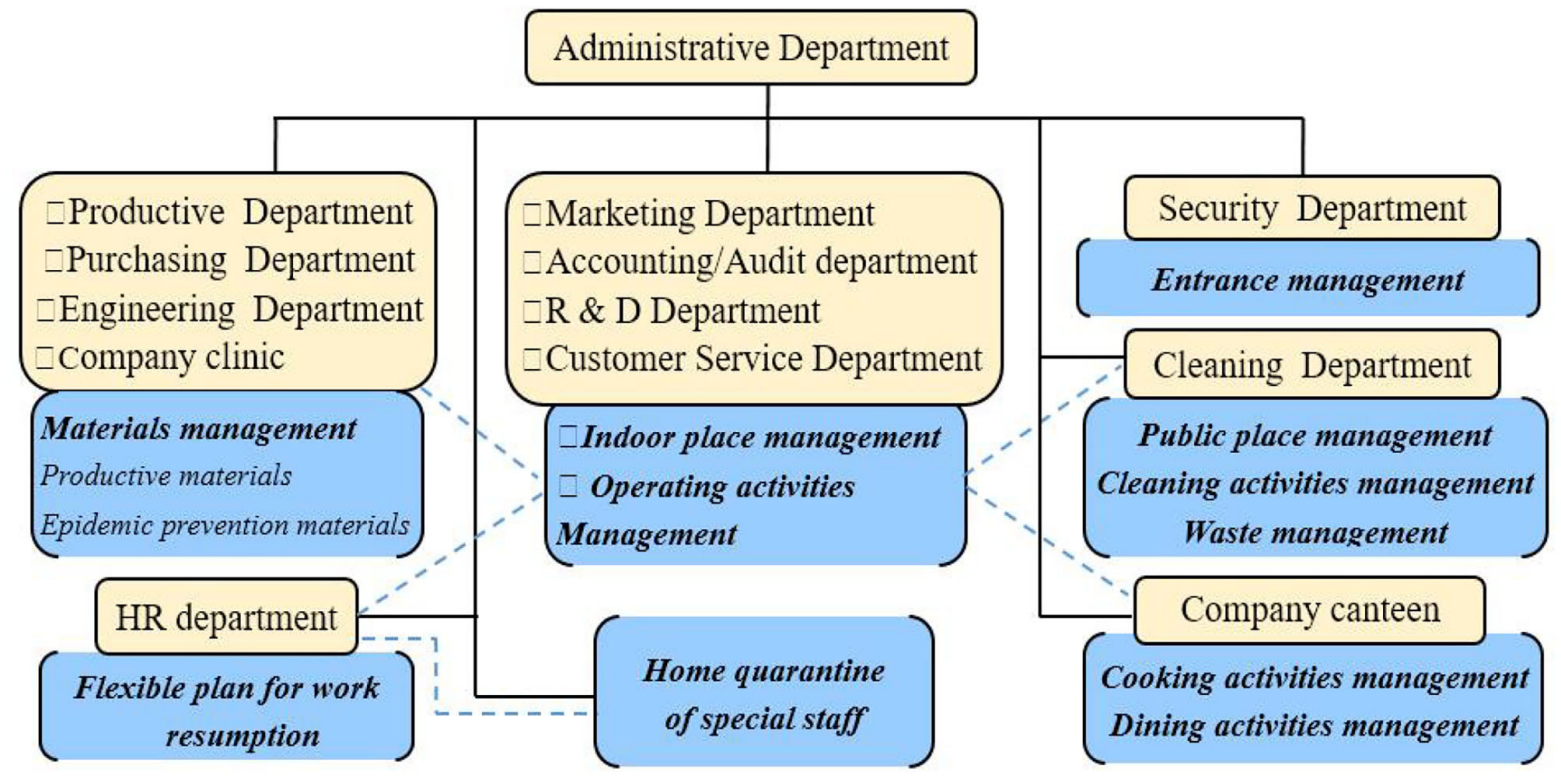
Divisions of various departments in epidemic prevention.

In response to the spread of the epidemic, Company C set up COVID-19 epidemic prevention committee and took a range of measures to protect its employees from being infected. Epidemic prevention measures employed by Company C consist of the following parts. First, COVID-19 epidemic prevention committee in company C examined the government's guidance documents carefully, including “Notices on epidemic prevention during work resumption” (issued by Qingdao Municipal Government, February 6), “Opinions on Epidemic Prevention during Work Resumption” (issued by People's Government of Shandong Province, February 19) and the “Guidelines on Epidemic Prevention for Work Resumption of Enterprises and Institutions” (issued by People's Government of Shandong Province, February 21). Second, COVID-19 epidemic prevention committee in Company C tried to put forward prevention measures available for the company's actual situation based on the government's guidance documents. Third, COVID-19 epidemic prevention committee held 3 meetings to examine the feasibility of these countermeasures in depth, and made some refinements and proposed a detailed plan for epidemic prevention, mainly reflected in the following two documents, that is, “Management plan during Covid-19” and “Notices on dining service management during Covid-19 pandemic.” In order to improve the prevention measures and promote the epidemic prevention action, Company C clarified the responsibilities of each department during the epidemic prevention (Figure 1).

#### Health Education

Adequate knowledge of personal health and epidemic prevention is the basis for employees to prevent the epidemic at work. Company C conducted online health education related to the epidemic before work resumption. The scope of health education for the employees includes hand washing, drinking water, mask wearing, indoor ventilation, disinfection measures, health habits, and self-psychological adjustment. The main contents of the health education on are as follows:

1. *wear masks when you're out, try to take private cars, bicycles and other means of transportation instead of public transportation*.
2. *If you have to take public transportation, remember to wear a mask all the way and try to avoid touching the vehicle with your hands*.
3. *If you touch public goods during your trip, use alcohol to disinfection your hand if conditions permit, wash your hands in time when you arrive at home or the company, and fully disinfect your exposed belongings*.
4. *do not rub your eyes, nose, and mouth with water during going out*.
5. *You and your family members should try their best to avoid going to public places with large crowds. If you have to, remember to take the protective measures above*.

Source: Company C's internal website

(authorized by Company C 's administrative department)

### Facilities and Material Management During Work Resumption

Through analysis on the data from semi-structured interview and field research, it has been found that Company C's place and material management includes the following four aspects during the epidemic.

#### Entry/Exit Management

1. ***Entry/exit management*** “One entry, one exit” method was took to keep the company safe.
2. ***Entry/exit authorization*** Company C formulated different Entry/exit authorizations for different personnel, which could inhibit cross-infection of the COVID-19 epidemic in the company. An interview with the company's entry/exit manager can clearly show how Company C has implemented entry/exit authorization.
  *Our company formulated various entry/exit authorization for different types of personnel. If the visitors are the employees of our company, they will receive temperature monitoring to check whether their body temperature is lower than 37 degrees. If the employee's body temperature exceeds 37 degrees, he/she is forbidden to enter the company. Accordingly, we will report this employee's temperature information to the company's administrative department, and the employee will be required to stay at home for self-isolation for 14 days…*.
  *Source: interview with Mr. Zhu, the security manager*
3. ***Free mask distribution*** At the beginning of work resumption, Company C distributed free masks to its employees and visitors when masks were in short supply. With the increase of mask production in China, masks can be purchased from pharmacies at normal prices in April. After April 6, Company C no longer provide free masks for its employees and visitors, and require them to wear a mask to go inside and outside.
4. ***Hand Disinfection*** Company C's security guards were responsible for hand disinfection, which was offered to individuals entering the company.

#### Indoor Place Management

1. ***Indoor place disinfection*** Company C conduct daily disinfection in their offices, factories, canteens and other indoor places. For the offices, the ground is disinfected with 84 Disinfectant twice a day. Desktops and office equipment (such as telephones, computer screens, keyboards, mouse, calculators, printers, door handles, and window handles) should be disinfected twice a day. Notice that the power supply should be cut off when electronic equipment is disinfected. As for the factory, the central control room, the operation room, external operation room, duty room and other important areas are disinfected twice a day, and production tools such as machines and equipment are disinfected once a day. In terms of the canteen, the ground is disinfected with 84 Disinfectant three times a day, the tables and chairs are disinfected with alcohol-based disinfectant three times a day, and utensils are disinfected with ultraviolet and high temperature three times a day. Employees are responsible for the ventilation of the room where they work.
2. ***Indoor place ventilation*** Company C conducted ventilation for indoor places such as offices, factories, canteens three times a day for 20–30 min each time. Employees in different departments are responsible for the ventilation of their own spaces. Ventilation is carried out after disinfection.
3. ***Stopping the use of the central air conditioner*** Given central air conditioner may cause cross infection, Company C turned off the company's central air conditioner during the epidemic. And thus, independent air conditioners are used to regulate the indoor temperature.

#### Public Place Management

1. ***Frequent disinfection in public places*** Public places are disinfected frequently. An interview with the company's cleaning staff can show how public spaces were disinfected.
  *During the epidemic, our company increased the frequency of cleaning and disinfection in public places. Public places such as the corridors, stairs, halls, toilets and garages, should be disinfected twice a day with 84 Disinfectant. Public office spaces, such as conference rooms, reception rooms, recruitment rooms, lounge, also need to be disinfected twice a day. Escalators, door handles and other key positions are disinfected three or four times a day*.
  *Source: interview with Ms. Liu, the company's cleaning staff*
2. ***Putting hand sanitizer in the bathroom*** The cleaning department put hand sanitizer in the bathroom to help employees get rid of hand bacteria.
3. ***Stopping the use of the elevator*** The elevator is a closed space with high personnel density, which may increase the risk of cross-infection. Thus, Company C temporarily stopped the use of the elevator during the epidemic.

#### Management on Epidemic Prevent Materials, Productive Materials, and Waste Materials

1. ***Epidemic prevention materials*.** In response to the epidemic, the company's medical department have access to sufficient medical supplies for all employees, which include N95 masks, disinfectants, infrared thermometers, protective suits, antibiotic hand sanitizer, disposable gloves, and so on.
2. ***Raw material procurement*** During the epidemic, the procurement department optimized its procurement process and methods in order to reduce the risk of exposure to the virus. The following interview with the manager in the procurement department could illustrate the procurement process and methods during the pandemic.
  *During the epidemic, in order to reduce the risk of Virus infection during external procurement, we prefer to purchase the raw materials through online platforms, and the supplier will deliver the goods to us. We will go out to purchase raw materials when absolutely necessary. We will wear masks and gloves all the time when we go out to purchase raw materials. When we complete our outdoor procurement, we should register relevant information about our outdoor procurement, and receive temperature monitoring before entering the company. Raw material procurement both online and offline will be disinfected*.
  *Source: Mr. Chen, manager of the company's procurement department*
3. ***Waste materials*** The cleaning department disinfects and destroys garbage such as discarded masks and gloves, disposable plastic food containers, and manufacturing wastes as soon as possible, which could inhibit the spread of the virus within the company.

### Employee Activity Management During Work Resumption

Through the analysis of the data obtained from semi-structured interviews and field research, it has been found that Company C's employee activity management includes the following four aspects during the epidemic.

#### Operating Activities

1. ***Administrative activities*** Administrative employees are required to wear masks all the time after entering the company except for dining time, and should keep 1 meter distance from each other. Masks worn by employees should be disposable medical masks or N95 masks. Considering that group office activities are more likely to lead to cross-infection among employees. Company C suspended group office activities during the epidemic. The following interview with the manager of training and conference department could display administrative employee activities.
  *During the epidemic, in order to avoid cross-infection caused by close contact among our administrative employees, we completely stopped offline meetings, and switched to WeChat and Tencent for online meetings. In addition, we suspended gathering activities such as team building and collective workshops. In fact, what concerned us most is how to deal with visitors from other companies due to business needs. To deal with this issue, we not only require them to comply with our entry/exit management scheme, but also try our best to control the number of visitors and reduce the length of their visits*.
  *Source: interview with Ms. Wen, manager of training and conference department*
2. ***Productive activities*** Considering that the worker density in the factory is relatively high, workers in the factory are required not only to wear N100 masks but also disposable gloves and protective caps when they are working. In addition, private talks are not permitted during the production, and smoking is strictly prohibited in the any places in the factory during the epidemic. In order to lower the respiratory load of workers, the factory provided a flexible working time and an airy working environment for its workers. Besides, when workers took a break, they were allowed to go out of their workshops and take off their masks for taking a good breath under the condition that they should keep a physical distance between each other.
3. ***Business activities*.** At the beginning of work resumption, Company C suspended all its business trips and maintained its business negotiation through Internet. After mid-April, employees were allowed to make business travel, and were provided with sufficient masks and portable disinfectant. Notice that they were asked to report their personal health information every day.

#### Cleaning Activities

1. ***Cleaning process*** The cleaning department is responsible for cleaning and disinfecting all the public areas in the company. The company has made strict regulations on the cleaning process. The following interview with the cleaning staff could show the cleaning process in detail.
  *Before our everyday work, we need to receive body temperature measurement and hand disinfection from the company. Masks and gloves are required to wear during work. Of course, cleaning is a dangerous job during the epidemic, so we are required to take self-protection precautions during work. We should wear gloves and masks in the correct way. We wash and disinfect our hands and faces immediately after work. The company provides us with exclusive cleaning tools to prevent cross-infection*.
  *Source: Interview with Ms. Liu, cleaning staff*
2. ***Supervision scheme*** To ensure the quality of the cleaning work, the manager of the cleaning department would conduct post-inspection and temporary spot checks on the cleaning work.

#### Cooking Activities

1. ***Food Ingredients procurement*.** During the epidemic, the company's canteen standardized the purchasing process of food ingredients, and the details are as follows.
  *During the epidemic, we are strict with the purchasing process of food materials. The purchasing personnel are required to wear masks and disposable gloves when purchasing the food ingredients. They are also required to wash their hands, disinfect their clothing, wear masks, helmets, and disposable gloves before entering the canteen. After the purchasing work is completed, the purchasing personnel are required to wash and disinfect their hands immediately*.
  *Source: Interview with Mr. Qu, head chef of the canteen*
2. ***Cooking process*.** Chefs are required to wear masks, protective caps and disposable gloves during cooking. All food must be disinfected at high temperatures.
3. ***Diet menu improvement*.** Raw and cold food are temporarily canceled and the proportion of vegetables was increased, which could improve the immunity of employees. Poultry meat and eggs were prohibited.

#### Dining Activities

During the epidemic, Company C took innovative maneuvers regarding the management on employee dining activities.

1. ***Packed lunch*** Employees had meals together in the canteen before the pandemic. If the company continues this dining model, there would be an increased risk of cross-infection. To deal with this challenge, Company C changed the dining model. The canteen packs a lunch for every employee and distributes them to the employees, which could reduce the risk of cross-infection.
2. ***Dining at staggered times*** Employees are allowed to take packed lunches at staggering times. The specific dining time was assigned to each department.
3. ***Scattered dining*** Employees who get the packed lunch should return to their offices and have meal at their own offices. Safe distance should be kept during dining time.
  *I think it is necessary for our company to adopt this dining method. The spread of COVID-19 pandemic is always in a diversified and hidden way, and we don't know who carries the virus. This dining method could avoid close contact among employees and inhibit the spread of the virus in the company. For example, as sale staff, we would contact many people during their business trips, when we come back to the company, we must pay more attention not to contact with other employees. Although this dining method is somewhat inconvenient, it is completely acceptable. The epidemic prevention requires everyone's joint efforts, which could benefit his/her own health as well as others' health*.
  *Source: Interview with Mr. Tong, staff of the marketing department*

#### Home Quarantine

At the beginning of China's work resumption, many enterprises experienced cluster infections and one of the reasons is that some employees returning from the most affected provinces did not receive home or centralized quarantine. Therefore, the central government of China issued the *Guidelines on measures for epidemic prevention and control for work resumption by enterprises and institutions* in February 21, requiring “the personnel returning from areas most affected by Covid-19 epidemic to receive home or centralized quarantine for medical observation.” Under the guidance of national policies, Company C formulated its own home quarantine plan for its employees.

*In view of the persistence of the epidemic, our company has implemented different home quarantine plans for different types of employees. Firstly, for the employees whose body temperature is higher than 37 degrees and those returning to Qingdao from provinces other than the epidemic epicenter provinces, they are required to receive home quarantine for 14 days before they can enter the company to work. Secondly, for the travel personnel returning to Qingdao from provinces other than the epidemic epicenter provinces, our company will subsidize them to get nucleic acid testing and receive home quarantine for 14 days before they can enter the company to work. Thirdly, for the employees returning to Qingdao from epidemic epicenter provinces, our company subsidize them to receive nucleic acid testing and let them work at home during the epidemic. All the employees staying at home are required to report their body temperature daily*.

*Source: interview with Ms. Ma, head of administrative office*

## Discussion

In order to understand the company's experience of epidemic prevention in China, we take Company C as a typical case and obtain the practical data by field research and semi-structured interviews. In this study, we try to identify the practices Company C took to conduct epidemic prevention during work resumption. Company C's measures include the following three aspects, that is, work resumption preparation, facility and material management, and employee activity management. The research findings in this study could not only contribute to fighting COVID-19 epidemic at present, but also help the company to cope with future epidemic and other public health emergencies.

Most of Company C's measures on epidemic prevention are technical and practical aspects without political orientations, which could be applied to other countries. For instance, health education, a flexible work resumption, entry/exit management, indoor management, public place management, materials and waste management, as well as management on office/production activities, cleaning, cooking, and dining are technical measures on epidemic prevention. However, there are still some preconditions that affect what and how companies to deal with the epidemic.

The first precondition is social culture, especially the privacy standards and freedom consideration. For example, Company C requires all its employees to report their relevant information before work resumption, which covers health status, travel routes, contact objects and so on. This measure would be accepted by East Asian countries such as China, Japan, and Singapore (34, 35), but may be considered as infringement of privacy in Western countries (36). In addition, Company C requires employees returning from the frontline of the epidemic (Hubei province) to perform home quarantine. However, forcing employees to perform home quarantine may be considered as a restriction on personal freedom in Western countries. In France, Britain, Germany and the United States, there are demonstrations against home quarantine and home isolation. Therefore, personnel information investigation and home quarantine should be treated cautiously when applying them to other countries and regions.

The second precondition is national/local OSH-regulation and temporary guideline for epidemic prevention. The epidemic prevention practices taken by Company C were based on the national OSH-regulations. In addition, the epidemic prevention practices taken by Company C were also guided by the local regulations on epidemic prevention. However, different countries have different OSH-regulations, which could provide a guideline for their companies' epidemic prevention and bring important impact on their prevention actions (37, 38). Temporary prevention guideline or suggestion issued by the government during the epidemic may also provide reference for the company's prevention practices (39, 40). These are the key factors companies need to consider for epidemic prevention.

The third precondition is at the company level, including OSH practice, resources, and physical environment. In the countries where companies have conducted OSH practice, epidemic prevention measures were not created from scratch, but are an improvement on the existing OSH program (41, 42). During COVID-19 epidemic, Company C formulated its epidemic prevention plan based on its existing OSH practices and epidemic prevention experiences from fighting SARS in 2003. Medical resources is another factor which has an important impact on a company's epidemic prevention practices, which is the basis for the epidemic prevention. Physical environment is also an important factor, which exerts a great influence on the company's epidemic prevention. The physical environment of the company may vary according to the attributes of the company. Thus, prevention measures such as environment ventilation, disinfection, physical distancing need to be implemented according to company's specific physical environment (43).

The fourth precondition is the influence of modern technology. After the outbreak of the COVID-19 epidemic, Company C scheduled new working modes, which was based on the development of modern technology, especially the emergence of technologies of telework and online communication. For instance, online meetings and remote work were introduced. In fact, the emergence of “work from home” during COVID-19 epidemic is based on IOT (Internet of Things) technologies (44, 45). Modern technologies does provide more possibilities for the company's epidemic prevention.

In terms of research design, a single-case study method is used to investigate epidemic prevention measures taken by a Chinese company in the context of COVID-19 epidemic. Although single-case study method is widely used and has been considered as applicable (46), it still cannot provide a representative picture of common practice related to the research question, and thus encounters insufficiency on producing knowledge that transcends the case in question (47). This is the common limitation of all single-case studies (48).

## Conclusion

This paper takes Company C as a typical case, and uses field research method to summarize the practice and experience of the company's epidemic prevention and control comprehensively. Company C's measures include the following three aspects, that is, work resumption preparation, facility and material management, and employee activity management. Specifically, work resumption preparation could build a good basis for making thorough resumption and epidemic prevention plan. Facilities and material management could block the chain of epidemic spread through the physical environment. Specifically, entry/exit management could prevent the invasion of the pandemic within the company; indoor management and material and waste management could prevent cross-infection in the company. Employee activity management could block the chain of epidemic spread through the interpersonal communication. In this respect, work from home could avoid the cross-infection within the company. Also, operating management, cleaning management, cooking and dinning management could prevent cross-infection in the company. In summary, the epidemic prevention measures mentioned above cover all places in the company where the epidemic could potentially spread, and block the chain of epidemic spread from every aspect of employee activities. The research findings could shed some light on how companies in Western countries resume work and production during the COVID-19 pandemic.

## Paper Context

Promoting industry and business to resume work safely has become an important step to be taken by all countries Chinese enterprises have taken multiple measures to prevent the spread of the COVID-19 virus during work resumption. This study aimed to explore epidemic prevention measures a Chinese company (Company C) took during work resumption. The research findings could shed some light on how companies in Western countries resume work and production during the COVID-19 pandemic.

## Data Availability Statement

The raw data supporting the conclusions of this article will be made available by the authors, without undue reservation.

## Ethics Statement

The studies involving human participants were reviewed and approved by this study received approval from the Public Management research ethics committee from Ocean University of China. The patients/participants provided their written informed consent to participate in this study. Written informed consent was obtained from the individual(s) for the publication of any potentially identifiable images or data included in this article.

## Author Contributions

All authors listed have made a substantial, direct and intellectual contribution to the work, and approved it for publication.

## Conflict of Interest

The authors declare that the research was conducted in the absence of any commercial or financial relationships that could be construed as a potential conflict of interest.

## Footnotes

^1^These employees went on a business trip from December 2019 to January 2020, when the epidemic had already broken out in Hubei province with Wuhan as the epicenter. However, China and the public did not have a full understanding of the epidemic during this time, so the company still arranged sales staff to travel to the above locations during this period.
